# A commentary on “Epineurectomy of extracranial facial nerve trunk for non-flaccid sequelae following Bell’s Palsy: a single-arm trial”

**DOI:** 10.1097/JS9.0000000000003342

**Published:** 2025-09-08

**Authors:** Yupeng Guo, Xuanwei Dong, Guoqiang Chen

**Affiliations:** Department of Neurosurgery, Aviation General Hospital, Beijing, China

*Dear Editor*,

We read with great interest the recent article titled “Epineurectomy of extracranial facial nerve trunk for non-flaccid sequelae following Bell’s palsy: a single-arm trial”^[1]^ published in the *International Journal of Surgery*. This study aims to evaluate the safety and effectiveness of a novel surgical procedure – epineurectomy of the extracranial facial nerve trunk – as a possible treatment. The findings demonstrated that non-flaccid facial palsy sequelae can be safely and successfully alleviated by epineurectomy of the extracranial facial nerve trunk. Future studies must, however, address a number of important concerns.

First, despite being a widely used scale for assessing facial paralysis, the Sunnybrook Score, the main endpoint, has significant subjectivity and is unclear in terms of inter-rater consistency^[2]^. Regarding secondary endpoints, the improvement in “eye fissure narrowing” showed limited efficacy, though the authors failed to explore underlying causes. Moreover, the study excluded comprehensive quality of life (QoL) assessments such as the SF-36, relying solely on the Facial Disability Index (FDI), which may overlook critical dimensions like mental health. Future research should integrate objective indicators (e.g., electromyography) with diversified QoL tools to comprehensively evaluate surgical outcomes.

Second, the study’s 12-month follow-up period made it hard to evaluate the stability of surgical results over the long run^[3]^. Postoperative sequelae of non-relaxation facial paralysis are typically chronic conditions, and short-term improvements may not reflect long-term efficacy. For instance, previous studies mentioned recurrence after 2–3 years with alternative surgical methods (such as selective nerve section), but this study did not provide similar data. Additionally, two cases of incomplete recovery from adverse events like incision numbness remained at 12 months, suggesting that longer follow-up could reveal more issues. Future research is recommended to extend follow-up to 3–5 years and evaluate recurrence rates.

Third, although the author reported mild side effects including discomfort and numbness from the incision, no comprehensive information was given about the prevalence or treatment of serious side effects (such irreversible damage to the facial nerve). The procedure involves the main trunk of the facial nerve, which carries high risks. However, the study only reported 10 cases of temporary postoperative facial paralysis exacerbation that all resolved, with no documented instances of failed intraoperative nerve monitoring or corresponding management strategies. Additionally, the long-term compatibility and effectiveness of the artificial nerve sheath (GI 5 × 30) remain unassessed. Future research should establish a standardized complication classification system (e.g., Clavien-Dindo) and provide more comprehensive safety analyses.

This study preliminarily demonstrates that facial nerve main trunk outer membrane resection can improve non-relaxation sequelae following Bell’s palsy. However, it has limitations including design flaws (single-arm, small sample size), short follow-up period, and single outcome measure. We recommend conducting a future multicenter randomized controlled trial with extended follow-up duration, integration of objective indicators, and deeper exploration of the underlying mechanisms. All of this was noted in relation to the TITAN guideline, which calls for transparency in the reporting of artificial intelligence^[4]^.

## Data Availability

No data was used in this Letter to the Editor.
